# Targeting the Ghrelin Receptor as a Novel Therapeutic Option for Epilepsy

**DOI:** 10.3390/biomedicines10010053

**Published:** 2021-12-27

**Authors:** An Buckinx, Dimitri De Bundel, Ron Kooijman, Ilse Smolders

**Affiliations:** 1 Research Group Experimental Pharmacology, Department of Pharmaceutical Chemistry, Drug Analysis and Drug Information, Center for Neurosciences (C4N), Vrije Universiteit Brussel (VUB), 1090 Brussels, Belgium; an.buckinx@vub.be (A.B.); dimitri.de.bundel@vub.be (D.D.B.); 2 Research Group Experimental Pharmacology, Center for Neurosciences (C4N), Vrije Universiteit Brussel (VUB), 1090 Brussels, Belgium; ron.kooijman@vub.be

**Keywords:** epilepsy, ghrelin, ghrelin receptor

## Abstract

Epilepsy is a neurological disease affecting more than 50 million individuals worldwide. Notwithstanding the availability of a broad array of antiseizure drugs (ASDs), 30% of patients suffer from pharmacoresistant epilepsy. This highlights the urgent need for novel therapeutic options, preferably with an emphasis on new targets, since “me too” drugs have been shown to be of no avail. One of the appealing novel targets for ASDs is the ghrelin receptor (ghrelin-R). In epilepsy patients, alterations in the plasma levels of its endogenous ligand, ghrelin, have been described, and various ghrelin-R ligands are anticonvulsant in preclinical seizure and epilepsy models. Up until now, the exact mechanism-of-action of ghrelin-R-mediated anticonvulsant effects has remained poorly understood and is further complicated by multiple downstream signaling pathways and the heteromerization properties of the receptor. This review compiles current knowledge, and discusses the potential mechanisms-of-action of the anticonvulsant effects mediated by the ghrelin-R.

## 1. Introduction

Epilepsy is a neurological disease characterized by spontaneous and recurrent seizures [1]. With approximately 50 million patients, it is one of the most common neurological diseases worldwide [2]. Despite the availability of a wide range of antiseizure drugs (ASDs), up to 30% of patients suffer from pharmacoresistant epilepsy [2], of which a large proportion has temporal lobe epilepsy (TLE) [3,4]. This highlights the urgent need for the development of novel pharmacological treatment options.

One of these potential options is the orexigenic peptide, ghrelin. Ghrelin exerts both peripheral as well as central effects, and is primarily secreted by X/A-like cells in the stomach [5], but also, to less extents, in the small intestine, kidney, testis, pancreas, and the brain [5,6,7,8,9,10]. Peripherally, ghrelin plays an important role in gastric acid secretion, gastric emptying, and gastric motility [11,12,13], and it maintains glucose homeostasis via the inhibition of the insulin response to glucose administration [14]. Additionally, ghrelin is generally accepted to be a cardioprotective peptide [15,16].

In the central nervous system (CNS), ghrelin and its receptor are best known for their critical role in food intake, mediated by neuropeptide Y and agouti-related peptide [17,18,19] (reviewed in [20]). Additionally, ghrelin confers a regulatory role on growth hormone (GH) release [19], is implicated in learning and memory [21,22,23], modulates motivation and reward [24,25], and regulates the stress response (reviewed in [26]).

Soon after its discovery in 1999 [27], the interest in ghrelin within the context of epilepsy started to emerge. Ghrelin levels were shown to be altered in epilepsy patients, and ghrelin administration in preclinical seizure and epilepsy models is considered to be anticonvulsive [28,29,30,31]. However, up until now, the exact mechanism-of-action remains to be understood.

## 2. Ghrelin and Its Receptor

The main molecular form of ghrelin is a 28-amino acid (AA)-long peptide, with the active form containing a unique acylation on serine 3 [32]. Ghrelin is transcribed as a 117-AA-long preproghrelin. This is cleaved to render proghrelin, after which it undergoes acylation on serine 3, established by the membrane-bound enzyme, ghrelin O-acyltransferase (GOAT), which is distributed in a similar manner to ghrelin [33,34]. The acylation is either an octanoylation (eight-carbon fatty acid) or decanoylation (ten-carbon fatty acid) [33,34]. This action is followed by further processing of the 94-AA-long acylated pro-ghrelin by prohormone convertases 1/3 (PC1/3), which results in acylated ghrelin (AG), and also yields the mature peptide, obestatin [35]. Acylation on serine 3 was first believed to be imperative for the ability of ghrelin to bind to its receptor and to exert ghrelin’s biological function [27]. Later, it became clear that desacyl ghrelin (DAG) is not completely devoid of physiological actions, as it was shown to also induce food intake, albeit through orexin neurons and not ghrelin receptor (ghrelin-R)-expressing neurons [36]. On the other hand, the anticonvulsant effects elicited by DAG required the presence of the ghrelin-R [37]. DAG shares some physiological functions similar to ghrelin but antagonizes others. Therefore, opposite effects might be mediated via a distinct receptor, and similar effects may be mediated by the ghrelin-R [38].

In human plasma, circulating esterases deacylate ghrelin, and 90% of total ghrelin consists of DAG, while only 10% consists of acylated ghrelin [39]. Ghrelin is rapidly cleared from plasma, with a plasma half-life ranging from 9–13 min for ghrelin, and 27–34 min for total ghrelin, including DAG [40,41]. Although the plasma concentration of DAG is much higher than that of ghrelin, its binding capacity to the ghrelin-R is substantially lower compared to ghrelin [38,42], which may explain why DAG was initially considered to be the “nonactive” variant of the peptide.

Recently, GOAT was shown to be expressed on the cell surface of mature bone marrow adipocytes, and to be necessary for DAG to promote adipogenesis in mice [43]. In line with this observation, GOAT was shown to be localized in the hilar border of dentate gyrus (DG) in the hippocampi of mice, and the incubation of live hippocampal slice cultures with DAG showed equal binding to the ghrelin-R as incubation with ghrelin, reliant on both the ghrelin-R and GOAT expression [44]. These data suggest that the local reacylation of DAG via GOAT expression at the cell surface may occur, and that it may be relevant for the biological functions of DAG mediated via the ghrelin-R.

### 2.1. Signaling Pathways and Heteromerization Complicate Ghrelin-R Signaling

Ghrelin establishes its numerous effects by interacting with its G-protein-coupled receptor (GPCR), of which two isoforms exist: the full-length (366 amino acids (AA) long) 7-transmembrane GPCR GHSR1a (a growth hormone secretagogue receptor, denoted as “ghrelin-R”), and a shorter (289 AA long) 3′-truncated variant, GHSR1b [27,45]. This nonsignaling short variant lacks the ability to exert biological effects in response to ghrelin and hampers the cell surface expression of the functional GHSR-1a variant, thus acting as a coregulator of ghrelin-R signaling [46,47].

The ghrelin-R is present in the brain and the periphery. The peripheral sites of ghrelin-R expression include the pancreas, spleen, bone tissue, cardiac tissue, the thyroid gland and immune cells, the adrenal glands, adipose tissue, and the vagal afferents [45,48].

Centrally, the ghrelin-R is widely expressed in a variety of brain areas and shows high expression levels in several nuclei of the hypothalamus, among which are the arcuate nucleus and the anterior hypothalamic nucleus. The receptor is further expressed in the olfactory bulb, the neocortex, in a variety of nuclei in the midbrain, in the pons, and in the medulla oblongata. These include the globus pallidus, the area postrema, the nucleus tractus solitarius, the substantia nigra, and the ventral tegmental area. In the hippocampus, the ghrelin-R was shown to be modestly expressed in the Cornu Ammonis (CA)1, compared to the higher expression levels in CA2, CA3, and DG of the mouse brain [45,49,50,51].

The expression of the ghrelin-R is highly dynamic, and depends on the developmental stage [52], the disease states [53,54], the metabolic state of the organism [55], or ghrelin availability [49]. Additionally, the receptor has a rich molecular pharmacology, with a multitude of signaling pathways associated with the receptor, and an ability to alter canonical ghrelin-R signaling via the formation of functional heteromeric complexes with other receptors. These factors contribute to diverse ghrelin-R signaling patterns.

**Figure 1:**
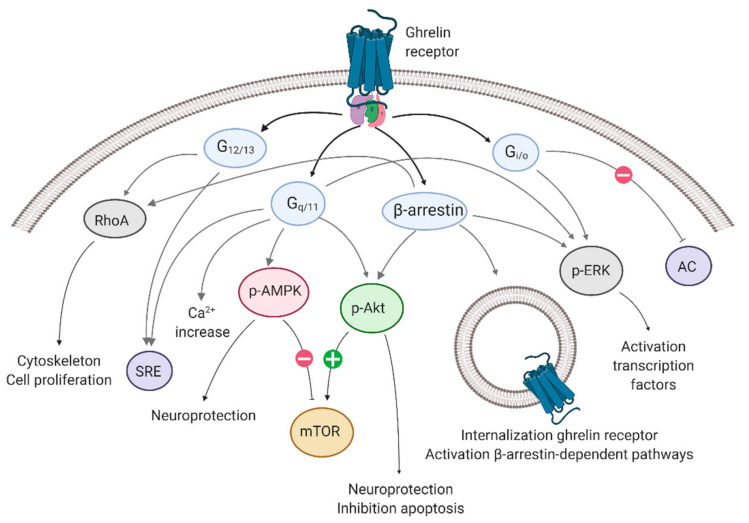
Signaling pathways associated with the ghrelin receptor. The ghrelin receptor employs Gα_q/11_ signaling, Gα_i/o_ signaling, and Gα_12/13_ signaling, followed by β-arrestin recruitment. Each G-protein/β-arrestin is associated with physiological effects. AC: adenylyl cyclase; AMPK: adenosine-monophosphate-activated protein kinase; ERK: extracellular signal-regulated kinase; mTOR: mammalian target of rapamycin; p-: phosphorylated-; RhoA: Ras homolog family member A; SRE: serum response element. Created with BioRender.com.

The signaling pathways downstream of the ghrelin-R include Gα_q/11_, Gα_i/o_, and Gα_12/13_ signaling, followed by β-arrestin recruitment [56,57,58]. The canonical Gα_q_ protein activates the phospholipase C (PLC)—inositol 1,4,5-triphosphate (IP3)—diacylglycerol (DAG) pathway, which leads to an intracellular calcium (Ca^2+^) increase [59,60]. Gα_i/o_ activates phosphatidylinositol-3-kinase (PI3K) [61] and reduces cyclic AMP (cAMP) levels via reduced adenylyl cyclase (AC) activity [60]. Gα_12/13_ signaling is associated with the activation of Ras homolog family member A (RhoA), other Rho guanine exchange factors, and their associated Rho kinases [62] (reviewed by [63]). Finally, G-protein-mediated signaling is halted via the recruitment of β-arrestin to the receptor [64], which not only vouches for the desensitization and internalization, but may also activate G-protein independent signaling pathways (reviewed in [65,66]). Recent studies have shown the ability of the β-arrestin-mediated activation of ERK1/2, mitogen-activated protein kinase (MAPK), the Akt/protein kinase B (PKB) pathways, and RhoA signaling [56,61,67] (Figure 1).

The ghrelin-R confers extraordinarily high intracellular signaling in the absence of ghrelin or a ghrelin-R full agonist, signaling at approximately 50% of its maximal capacity [67,68]. Constitutive activity includes signaling via G-proteins, while it does not entail β-arrestin-mediated endocytosis [67,68,69].

### 2.2. What Is Known about Ghrelin’s Central Availability?

Ghrelin, DAG, or synthetic compounds must access the brain to be centrally active. The transport of ghrelin across the blood–brain barrier (BBB) has been shown to occur via saturable mechanisms in mice [23,70], and does not depend on the expression of the ghrelin-R [71]. The notion that the BBB may be compromised in epilepsy should be taken into account, which would facilitate the availability of ghrelin to the CNS.

Additionally, systemically injected ghrelin was shown to cross the fenestrated capillaries in the circumventricular organs (CVO) via passive diffusion, and dose-dependently impacted more distant brain areas [72]. Finally, fluorescent ghrelin was shown to internalize in ependymal cells located in the choroid plexus and in β-type tanycytes, which constitute the foundation of the blood–cerebrospinal fluid (CSF) barrier (BCSFB). Fluorescent ghrelin was detected in periventricular hypothalamic tissue, and decreased with distance from the third ventricle [73]. The transport of ghrelin via the BCSFB depends predominantly on the presence of the ghrelin-R [74]. The kinetics of diffusion into the brain via the BCSFB is somewhat slower compared to the diffusion via the CVOs, with the CSF ghrelin concentrations peaking approximately 30 min after the ghrelin plasma concentration peak, depending on plasma ghrelin levels [75]. Additionally, an in vitro study showed that ghrelin was internalized in rat primary tanycytes via clathrin-coated vesicles [76].

Up until now, it has remained incompletely understood whether ghrelin is centrally available in areas more remote from the aforementioned barriers. It is possible that circulating ghrelin reaches certain permeable parts of the brain, and affects other areas indirectly, via the innervation of the nuclei located in the vicinity of accessible brain parts. This was shown in the case of the area postrema, which directly notes alterations in plasma ghrelin levels and innervates the nucleus tractus solitarius [72]. Additionally, central ghrelin expression may serve as an explanation for the high ghrelin-R expression in brain areas that are seemingly inaccessible to circulating ghrelin [9,27]. Indeed, central ghrelin messenger ribonucleic acid (mRNA) expression and immunoreactivity have been shown in multiple studies; however, there are also some studies refuting this notion (reviewed in [77]).

## 3. Studies in Humans

In humans, the majority of total circulating ghrelin consists of DAG, due to the deacylation of AG [39]. The acylation is located at the N-terminal part of the peptide, while the rest of the molecule is equivalent between AG and DAG. The studies outlined below do not always specify the portion of the peptide that is recognized by the used assays, nor is this information always available on the manufacturer’s website. Failing to specify which isoform was measured may explain some of the observed interstudy variations. Most of the studies investigating AG or DAG levels assessed this peptide in plasma (in a small number of studies, the saliva and urine ghrelin levels were also assessed) after overnight fasting and were conducted in children and adolescents. Ghrelin levels are negatively correlated with age [78], and even pubertal children have significantly lower total plasma ghrelin levels compared to prepubertal children [79]. Therefore, in this review, a distinction is made between studies on adults and studies on children.

### 3.1. Adults

Up to now, there has been no general consensus regarding the differences in interictal ghrelin levels between adult epilepsy patients and healthy subjects. Three studies showed lower ghrelin levels in seizure-controlled epilepsy patients compared to healthy controls [80,81,82], while two studies did not detect differences in plasma ghrelin levels between epilepsy patients and controls [83,84]. One study demonstrated that patients with seizure-controlled epilepsy had significantly higher serum ghrelin compared to healthy controls [85]. Three studies demonstrated that patients suffering from focal epilepsy had higher ghrelin plasma levels compared to patients suffering from generalized seizures [80,81,85]. Two studies were not able to replicate this finding [84,86] (Table 1).

The AG/DAG ratio was significantly higher in epilepsy patients compared to controls, and it did not differ between different epilepsy types, or between refractory and nonrefractory epilepsy [84]. In females with Rett syndrome, the AG/total ghrelin ratio was significantly increased compared to epilepsy patients not diagnosed with Rett syndrome [87]. An assessment of the ratios of AG and DAG or total ghrelin may explain the difficult-to-reconcile observations in the ghrelin levels, and may appear as a good alternative read-out for these studies. However, measuring AG from plasma is technically challenging, and the best practices for handling AG plasma samples are, up to now, not entirely resolved [88]. Thus, the sensitivity of AG to sample handling can lead to large observed interstudy variations.

To elucidate the impact of ASD treatment or epilepsy disease progression on ghrelin levels, some studies have assessed the interictal ghrelin plasma levels before and after ASD treatment. After two years of successful valproic acid treatment, only patients that had developed obesity had significantly lower plasma ghrelin levels compared to controls, while this was not the case in patients that had not developed obesity [83]. The serum DAG levels did not differ after three months of ASD treatment, while the AG levels were decreased after three months [80]. Finally, ASD-responsive patients had increased ghrelin levels compared to nonresponders in two studies, but not in another study [82,86,89]. A significant positive correlation has been shown between both the AG and DAG levels and disease duration, which could be indicative of ghrelin resistance, but could also be related to ASD use [84].

One study assessed the alterations in plasma ghrelin immediately after seizures. The AG and DAG levels decreased as soon as five minutes after a generalized seizure, and were restored after 24 h [89]. Moreover, in the preclinical pentylenetetrazole (PTZ) model, AG, but not DAG, as well as total ghrelin plasma levels, decreased 30 min after the induction of a seizure (see further) [90]. Overall, most studies show lower ghrelin levels in patients with epilepsy compared to healthy controls, or a decrease in ghrelin levels after a seizure.

### 3.2. Children

The latter statement could be extrapolated to children, as total ghrelin levels were significantly lower in prepubertal children with epilepsy compared to healthy controls [91,92]. Another study assessed the AG and DAG plasma levels within six hours after a seizure in children not yet receiving treatment (pretreatment), three months after treatment (post-treatment), and in healthy controls. The AG levels were significantly lower in the pretreatment group compared to the post-treatment group and the controls [93]. DAG levels were significantly higher in the post-treatment group compared to the pretreatment group in urine and saliva, but not in serum [93] (Table 2). Within the epilepsy group, lean children on valproic acid had significantly higher total ghrelin plasma levels compared to children on carbamazepine [91], but not compared to children receiving topiramate [94].

The majority of studies that assessed ghrelin levels related to disease progression did not detect significant differences between ghrelin levels measured over time (Table 3) [95,96,97]. One study showed that plasma ghrelin was significantly decreased after the initiation of valproic acid treatment in pubertal children, but not in prepubertal children, nor in children on oxcarbazepine. In the latter case, this may be due to the increased weight gain in the children receiving valproic acid [98].

#### The Ketogenic Diet

The ketogenic diet (KD) is an alternative treatment option for refractory epilepsy and has often been proven useful, particularly in children. It remains to be elucidated to what extent the alterations in AG or DAG may mediate some of the effects of the KD [104]. Both AG and DAG levels were shown to be decreased after the initiation of a KD in children with drug-resistant epilepsy [101]. Another study showed that AG plasma levels were decreased as soon as 30 days after the initiation of a KD in children with pharmacoresistant epilepsy [102]. One study did not detect alterations in ghrelin levels after the onset of a KD in drug-resistant epilepsy patients [103] (Table 3).

## 4. Preclinical Evidence for Ghrelin as a Potential Antiseizure Drug

Ghrelin, in both its acylated and deacylated form, as well as synthetic ligands, have been studied in rodent seizure and epilepsy models. The majority of these studies focus on the administration of ghrelin or ghrelin-R ligands to modulate seizures or epilepsy, while only a few studies have assessed plasma ghrelin levels in these models.

### 4.1. Ghrelin in Seizure and Status Epilepticus Rodent Models

Both systemic and intrahippocampal ghrelin administration were anticonvulsant in the acute rat PTZ model [105,106,107,108]. A longer pretreatment of 10 days with ghrelin elicited the same antiseizure effect [107]. One study showed that ghrelin administration in PTZ-treated rats enhanced cognitive capacity in terms of spatial memory [109], which is interesting in light of cognitive impairments as important comorbidities of epilepsy [110].

AG, but not DAG, and total ghrelin plasma levels were decreased 30 min after the induction of a seizure in the PTZ model [90]. This decrease was confirmed in another study, where total ghrelin serum and brain levels were lower in rats after acute PTZ injection, but also after chronic PTZ kindling [111]. Finally, the brain tissue and plasma total ghrelin levels were decreased in mice that exhibited seizures, which were elicited after 24 h of fasting, followed by scopolamine administration [112].

In a study conducted on a rat penicillin model performed under anesthesia, only 1 µg, but not 2 µg of ghrelin administered 30 min after penicillin significantly lowered the spike frequency. These data imply that ghrelin might not follow a linear dose–response curve [113]. There is one study that recently demonstrated ghrelin administration to be proconvulsive in a WAG/Rij rat model presenting with absence seizures, as ghrelin increased the number of spike–wave discharges and the total seizure duration one hour after administration [114].

Ghrelin has been assessed in various status epilepticus (SE) models. However, given the short duration of these experiments, they only reflect the effects of ghrelin on the phenomenon of SE, and not on the subsequent chronic recurrent seizures. Ghrelin was not anticonvulsant in SE models in rats [31,115], except for one study [116], while ghrelin exerted anticonvulsant effects in a pilocarpine tail infusion mouse model and an intrahippocampal kainic acid (IHKA) mouse model [29,116]. One explanation for these observations could be the short timing of ghrelin administration prior to the stimulus (only 10 min) in the rat models. Additionally, the doses that were used in these studies varied highly. Interestingly, it appears that the choice of species may be involved as well, as ghrelin (both at a dose of 0.08 mg/kg and 1.8 mg/kg) was anticonvulsant in mice, but not in the pilocarpine or KA rat model at a dose of 1.5 mg/kg. On the other hand, the effects exerted by ghrelin may not be strong enough to interfere with the development of SE (Table 4).

Ghrelin’s deacylated form, DAG, was anticonvulsant in the IHKA rat model, the intracerebroventricular pilocarpine rat model, and the pilocarpine tail infusion mouse model [37,115] (Table 5). While this was not the case with ghrelin, the administration of DAG only 10 min prior to a pilocarpine or IHKA stimulus was anticonvulsant [115]. A possible explanation may rely on the fact that DAG has a faster transport rate across the BBB in mice compared to ghrelin [71].

Another possible explanation could be the presence of GOAT in the hippocampus, which may locally acylate extracellular DAG [44]. This leads to the compelling hypothesis that DAG may exert anticonvulsant effects via its superior brain availability compared to ghrelin, in combination with local acylation, to render AG in the hippocampus and exert anticonvulsant effects via the ghrelin-R. This mechanism would drastically improve ghrelin availability at such difficult-to-reach brain areas. Additionally, this hypothesis may fit into the notion that DAG was shown to require ghrelin-R expression to exert anticonvulsant effects [37].

All preclinical studies involving the effect of ghrelin on seizures or SE have been conducted in male rodents. There is one study that used female rats to investigate whether ghrelin administration differentially affects the incidence of seizures at various time points of the estrous cycle. The authors found that ghrelin was anticonvulsant during all phases of the estrous cycle; however, the effects were more outspoken during the luteal phase compared to the follicular phase [108].

### 4.2. Ghrelin Receptor Agonists

A large number of shorter ligands with binding affinity at the ghrelin-R have been synthetized, of which the ghrelin-R agonists, macimorelin, capromorelin, and hexarelin have been tested in animal models of seizures or epilepsy (Table 6).

The pseudotripeptide, macimorelin (H-Aib-(d)-Trp-(d)-gTrp-formyl, also known as “JMV-1843”) was first synthesized in 2003 [120], and it is currently on the market for the diagnosis of GH deficiency [121,122]. It is a full agonist of the ghrelin-R and has a longer plasma half-life compared to the endogenous agonist [122,123]. Our group showed that macimorelin was anticonvulsant in both the acute 6-Hz mouse model and fully 6-Hz-kindled mice through the ghrelin-R [124], and in a dopamine 1 receptor (D1R)-mediated mouse kindling model [125]. Macimorelin did not exert anticonvulsant effects in the SE pilocarpine rat model [31,115], but was anticonvulsant in the IHKA mouse model [126]. These studies differed in the dose, the timing of the administration, and the species used, which may explain these conflicting findings.

As ghrelin or macimorelin were shown to exert neuroprotective [28,29,30,31] and anti-inflammatory effects [31,121,122] in seizure models (see further), our group recently studied whether macimorelin was able to interfere with epileptogenesis. The prevention or attenuation of the development of epilepsy could drastically reduce morbidity and the socioeconomic costs associated with refractory epilepsy. However, we found that macimorelin was anticonvulsive, but not antiepileptogenic, in the IHKA mouse model [126].

Capromorelin is a ghrelin-R full agonist with a high affinity for its receptor [127], and it is currently FDA-approved for veterinary use for increasing food intake [128,129]. Capromorelin was intrahippocampally infused two hours prior to intrahippocampal pilocarpine infusion in rats and decreased the total seizure severity score [116].

The hexapeptide, hexarelin, was developed as a GH secretagogue prior to the discovery of ghrelin [130]. Its potential anticonvulsant effects were assessed in both the pilocarpine rat model and the IHKA rat model. While a low dose (0.33 mg/kg) was anticonvulsant in the pilocarpine rat model, the same administration regimen was not anticonvulsant in the IHKA rat model [115]. This once more underscores the variation between the models and the species used in the discovery of novel potential ASDs, and advocates for the use of multiple seizure or epilepsy models in the discovery of potential new ASDs.

### 4.3. Administration of Other Ghrelin Receptor Ligands

Neutral antagonists prevent the activation of a receptor by blocking the agonist binding to the receptor, but do not affect its basal constitutive activity. Three ghrelin-R antagonists have been investigated in a variety of epilepsy models, of which the neutral antagonist, JMV-2959, was without effects in the pilocarpine rat model, in acute 6-Hz- or fully kindled mice, and in the D1R-mediated kindling model [115,124,125]. The hexapeptide, EP-80317 (Haic-D-Mrp-D-Lys-Trp-D-Phe-Lys-NH2), was anticonvulsive in the pilocarpine SE model and the 6-Hz-kindled mouse model [115,131,132] (Table 6). Interestingly, resistance to the initial anticonvulsant effects of EP-80317 treatment were observed with seizure progression in the 6-Hz-kindling model [131,132]. Its anticonvulsant effects were shown to be dependent on the peroxisome-proliferator-activated receptor, S-gamma (PPAR-γ), presumably via the cluster of differentiation (CD)36 receptor [131,132]. By contrast, one recent study showed that the ghrelin-R antagonist, D-Lys-3-GHRP-6, induced spontaneous seizures in an amygdala-kindled rat model, [133].

Intrahippocampal infusion of the inverse agonists, A778193 and [D-Arg1, D-Phe5, D-Trp7,9, Leu11]-substance P, were anticonvulsant in the intrahippocampal pilocarpine infusion rat model. Inverse agonists are typified by their ability to block the intracellular signaling of a receptor, including basal constitutive signaling, which resembles an absence of the receptor. In line with this notion, ghrelin-R knock-out (KO) mice were shown to be protected from seizures [116,124], which suggests that the absence of ghrelin-R signaling is anticonvulsant. In agreement with this, the biased agonist, YIL671, a Gα_q_ and Gα_12_ selective biased ligand of the ghrelin-R that is not able to recruit β-arrestin, increased the seizure burden in the D1R-mediated kindling model [125].

**Table 6:** Overview of anticonvulsant effects of ghrelin-R ligands in experimental epilepsy models. D1R: Dopamine 1 receptor; i.v.: intravenous; i.h.: intrahippocampal; IHKA: intrahippocampal kainic acid; i.p.: intraperitoneal; KA: kainic acid; min: minute; pilo: pilocarpine; Ref: reference; SP: [D-Arg1,D-Phe5,D-Trp7,9,Leu11]substance P.

Compound	Dose	Administration Regimen	Anticonvulsant	Animal Model	Ref
**Agonists**					
Macimorelin	0.33 mg/kg	i.p 10 min prior to pilo	no	Pilocarpine i.p. rat model	[31,115]
Macimorelin	5 mg/kg	i.p., 20 min prior to stimulus	yes	Acute 6- Hzmouse model	[124]
Macimorelin	5 mg/kg	i.v. infusion	yes	Fully kindled 6-Hz mouse model	[124]
Macimorelin	5 mg/kg	30 min prior to SKF	yes	D1R-mediated kindling mouse model	[125]
Macimorelin	5 mg/kg	14 days, 2×/day	yes	IHKA mouse model	[126]
Capromorelin	0.01–10 µM	i.h. infusion 120 min prior to pilo	yes	Pilocarpine i.h. infusion rat model	[116]
Hexarelin	0.33 mg/kg	i.p. 10 min prior to pilo	yes	Pilocarpine i.p. rat model	[115]
Hexarelin	0.33 mg/kg	i.p. 10 min prior to KA	no	KA i.p. rat model	[115]
**Antagonists**					
EP-80317	0.33 mg/kg	i.p. 10 min prior to pilo	yes/no	Pilocarpine i.p. rat model	[115]
EP-80317	0.33 mg/kg	i.p. 10 min prior to KA	no	KA i.p. rat model	[115]
EP-80317	0.33 mg/kg	i.p. 10–15 min prior to stimulus	yes	6-Hz repeated mouse model	[131]
JMV-2959	0.33 mg/kg	i.p. 10 min prior to pilo	no	Pilocarpine i.p. rat model	[115]
JMV-2959	10 mg/kg	i.p. 20 min prior to stimulus	no	Acute 6-Hz mouse model	[124]
JMV-2959	10 mg/kg	i.v. infusion	no	6-Hz fully kindled mice	[124]
JMV-2959	5 mg/kg	i.p. 30 min prior to SKF	no	D1R-mediated kindling mouse model	[125]
D-Lys-3-GHRP-6	1–100 µg	i.c.v. 30 min prior to stimulus	no	Amygdala kindling rat model	[133]
**Inverse Agonists**					
A778193	0.01–10 µM	i.h. infusion 120 min prior to pilo	yes	Pilocarpine i.h. infusion rat model	[116]
SP	0.01–10 µM	i.h. infusion 120 min prior to pilo	yes	Pilocarpine i.h. infusion rat model	[116]
**Biased Agonists**					
YIL781	5 mg/kg	i.p. 30 min prior to SKF	no	D1R-mediated kindling mouse model	[125]

## 5. Molecular Mechanisms-of-Action

### 5.1. Mechanisms of Ghrelin’s Anticonvulsant Action

Ghrelin-R expression is dynamic and may be influenced by the presence of a disease state, or may depend on exposure to ghrelin [54,55,134], which are both relevant in the context of ghrelin administration in seizure and epilepsy models. Neither ghrelin nor pilocarpine altered hippocampal ghrelin-R mRNA expression in the pilocarpine rat model [28], while another group showed a decrease in the hippocampal ghrelin-R mRNA expression in pilocarpine-treated rats, which was restored upon ghrelin administration [30].

Hippocampal Akt signaling was decreased in a pilocarpine rat model, which could be restored by ghrelin administration [28,30]. Akt is a downstream target of the ghrelin-R, which can be activated both by Gα_q_ signaling and β-arrestin recruitment. The ghrelin-R antagonist, EP-80317, restored the increased hippocampal phosphorylation levels of the other canonical downstream target ERK in the 6-Hz mouse model [131].

Up until now, the exact signaling pathways responsible for anticonvulsant effects downstream of ghrelin-R have remained elusive, but a few possibilities exist, on the basis of previous findings. Not only ghrelin-R agonists, but also ghrelin-R inverse agonists exerted anticonvulsant effects, and ghrelin-R KO mice were protected from seizures [116,124]. The truncated ghrelin variant, ghrelin (1–5) amide, shows similar EC_50_ values compared to ghrelin with regard to the ghrelin-R signaling pathways, but is unable to internalize the ghrelin receptor, and was not able to exert anticonvulsant effects [116]. Because of these intuitively irreconcilable observations, a novel concept emerged, hypothesizing that the absence of the ghrelin-R on the cell surface was responsible for exerting ghrelin’s anticonvulsant effect [116]. We showed that a Gα_q_ and Gα_12_ selective biased ligand of the ghrelin-R, YIL781, increased seizure severity in a kindling model [125]. Given these observations, β-arrestin recruitment remains the most probable pathway involved in ghrelin-R-mediated anticonvulsive effects and it requires further investigation.

However, we cannot completely exclude that G-protein-dependent signaling may be required for ghrelin-R-mediated anticonvulsant effects, as Akt and ERK activation have been described [30,32,135]. However, this would not fit with the notions of ghrelin-R KO mice being protected from seizures, and inverse agonists exerting anticonvulsant effects; in these cases, there is no G-protein-dependent signaling downstream of the ghrelin-R. Finally, one could hypothesize that the possibility exists that the signaling pathways downstream of β-arrestin may be responsible for ghrelin’s anticonvulsant effects. Indeed, Akt and ERK are mediators that can also be activated via β-arrestin-dependent signaling. Nonetheless, and also here, the data obtained from the experiments with ghrelin-R KO mice and inverse agonists [116] suggest otherwise, and point towards an absence of signaling, which is imperative for ghrelin’s anticonvulsant effects.

### 5.2. Mechanisms of Neuroprotection

Ghrelin increased the number of surviving neurons in the CA1 and CA3 hippocampal regions in the pilocarpine rat model [28]. Pilocarpine reduced the apoptotic repressor, B-cell lymphoma 2 (Bcl-2), increased the proapoptotic member Bcl-2-associated X protein (Bax), and increased cleaved caspase-3, crucial in apoptosis. Ghrelin was able to restore these markers, and may thus exert neuroprotection through antiapoptotic effects [28].

This latter finding was confirmed in the i.p. KA mouse model, in which ghrelin decreased cleaved caspase-3 immunoreactivity in pyramidal CA1 and CA3 neurons, and restored neuronal loss in CA1 and CA3 [29]. Terminal deoxynucleotidyl transferase dUTP nick end labeling (TUNEL)-positive cells were abundantly present in vehicle-treated KA mice, but no TUNEL-positive cells could be observed in ghrelin-treated KA mice. All of the above described effects were dependent on ghrelin-R, as they were reversed by the concurrent administration of a ghrelin-R antagonist [29]. A study conducted by Zhang and colleagues confirmed the necessity of ghrelin-R availability in order for ghrelin to exert its neuroprotective effects [30]. Ghrelin significantly rescued neuronal cell loss in CA3, and inhibited cleaved caspase-3 activation, mediated via the phosphorylation of Akt [30].

A two-week-long administration of the ghrelin-R agonist, macimorelin, in the IHKA mouse model, exerted anticonvulsant effects on spontaneous recurrent seizures, but did not increase neuronal survival in the CA1, CA3, and DG of the hippocampus. This could be due to the omission of pretreatment, as the onset of the treatment commenced 24 h after SE induction, or due to the additional two-week wash-out in this study [126]. A lower dose of macimorelin, administered prior to SE, was found to increase neuronal survival and decrease apoptosis in the DG in the pilocarpine rat model, but not in CA1 [31]. Additionally, this study was able to demonstrate neuroprotective effects exerted by macimorelin, but not anticonvulsant effects against the pilocarpine-induced SE.

The possibility should be considered that ghrelin exerts neuroprotective effects via the activation of a variety of signaling pathways mediated through the employment of G-proteins, whereas the rapid and subsequent internalization via β-arrestin signaling may be responsible for anticonvulsant effects. Unraveling which ghrelin-R downstream signaling pathway is responsible for a particular effect may be obtained by doing further experiments in genetic models, or by using biased ligands that selectively activate a subset of pathways while leaving others untouched.

### 5.3. Inflammation

Inflammation is a major hallmark of epileptogenesis, seizures, and chronic epilepsy. This ranges from infiltration of the inflammatory cells and the release of proinflammatory mediators to widespread gliosis [134,135,136,137]. Given the fact that inflammation is known to progress the development of epilepsy [138], one of the presumed mechanisms-of-action of ghrelin may rely on its ability to attenuate inflammation, stemming from both direct central actions, and through peripheral anti-inflammatory effects [139].

Ghrelin significantly reduced the elevated plasma calcitonin gene-related peptide (CGRP), substance P, interleukin (IL)-6, tumor necrosis factor (TNF)-α, and IL-1β in the PTZ rat model [117,118]. Additionally, ghrelin inhibited KA-induced increases in TNF-α, IL-1β and cyclooxygenase-2 (COX2) mRNA levels in CA1 and CA3 in the i.p. KA mouse model, mediated via the ghrelin-R [29]. Ghrelin restored KA-induced increased matrix metalloproteinase 3 levels, which is an important mediator of inflammation and neuronal cell death [29]. Additionally, KA-induced increases in microglia and glial fibrillary acidic protein (GFAP) immunoreactivity in CA1 and CA3 three days after SE were inhibited by ghrelin [29]. This was not detectable after a two-week wash-out following macimorelin administration in the IHKA mouse model, in which macimorelin was administered after KA, and not as a pretreatment [126]. Another study showed that ghrelin administration decreased cortical TNF- α and NF-κB expression in the pilocarpine rat model [140]. However, ghrelin did not alter serum levels of galanin, fibroblast growth factor (FGF-2), IL-6, TNF-α, and IL-1β in the Wag/Rij rat model with nonconvulsive absence seizures [114].

### 5.4. Oxidative Stress

Seizures induce oxidative stress, which, in turn, exacerbates seizures (reviewed by [141]). Ghrelin prevented the PTZ-induced decrease in the catalase activity in both the CNS and erythrocytes, and prevented the augmentation in thiobarbituric acid reactive substances levels, a measure for lipid peroxidation [142]. Additionally, ghrelin normalized superoxide dismutase levels, an enzyme responsible for clearing superoxide anion in the erythrocytes, brain, and liver [142]. These data suggest that ghrelin protects against oxidative stress caused by PTZ. It remains unclear whether the effects of ghrelin on decreasing oxidative stress are caused directly, or because ghrelin is anticonvulsant, and the lower number of seizures leads to decreased oxidative stress. However, in the WAG/Rij rat model with nonconvulsive absence seizures, ghrelin was not anticonvulsant, but it still reduced the malondialdehyde [114].

## 6. Functional Implications of Diminished Ghrelin-R Signaling in the Context of Excitability

While several molecular mechanisms-of-action have been described, it remains unknown how these contribute to the anticonvulsant effects of ghrelin, and how they lead to an overall decrease in the brain excitability. The heteromerization of the ghrelin-R with other receptors can lead to the preferential recruitment of other noncanonical signaling pathways [143]. Ghrelin, as well as other signaling molecules, may exploit this phenomenon for inducing ghrelin-R-mediated anticonvulsant effects.

Ghrelin-R activation results in intracellular Ca^2+^ increases through the canonical Gα_q_ protein [59,60]. One possible mechanism-of-action would be a decrease in intracellular Ca^2+^ in ghrelin-R-expressing neurons. Elevated levels of intracellular Ca^2+^ are associated with epileptiform activity and epileptogenesis [144,145]. Therefore, a reduction in intracellular Ca^2+^ may be an interesting putative mechanism for seizure suppression in the absence of ghrelin-R signaling. Various studies have shown the differential effects of ghrelin on neuronal excitability and synaptic transmission, which all support that ghrelin acts in a brain-region-specific manner [146,147,148].

The ghrelin-R is expressed in both excitatory neurons, as well as in inhibitory interneurons in the dorsal CA1. It was recently shown that a selective increased expression of the ghrelin-R in excitatory neurons was detrimental for learning and memory in mice, while an increased expression of the ghrelin-R in interneurons had a beneficial effect [149]. It remains to be uncovered if a dual effect on excitability also exists by, for instance, decreasing Gα_q_ signaling in excitatory neurons via β-arrestin, while increasing Gα_q_ signaling in inhibitory interneurons. Indeed, while GPCRs may be associated with several signaling pathways, these signaling pathways are not always all operative in the same cell. Thus far, the knowledge concerning the cell-specific expression of signaling pathways downstream of the ghrelin-R is lacking and requires further studies.

## 7. Conclusions and Future Perspectives

Ghrelin is increasingly recognized as a potential important player in seizures and epilepsy. Most studies show lower ghrelin levels in patients suffering from epilepsy, or lower ghrelin levels after a seizure. The exact implications of plasma ghrelin level alterations in epilepsy have remained, up until now, unknown, and should be further investigated in light of its treatments as well, including the KD. It is increasingly evident that there may be important differences between AG and DAG. This may advocate for future investigations of both isoforms of ghrelin in epilepsy, or for studying further whether the contributions of GOAT expression and the local reacylation of DAG are relevant for seizure control.

With only a few exceptions, ghrelin and synthetic agonists of the ghrelin-R are anticonvulsant in seizure, epilepsy, and SE models. The notion that both agonists and inverse agonists were anticonvulsant stirred up the discussion concerning the signaling pathways responsible for ghrelin-R-mediated anticonvulsant effects. The hypothesis that β-arrestin recruitment is involved should be more thoroughly investigated to confirm the relevance of this pathway. Overall, the complexity of ghrelin-R signaling, and the extensive list of other factors possibly influencing it, highlight the need for further investigations into the mechanism behind ghrelin-induced anticonvulsant effects.

## Figures and Tables

**Table 1:** Overview of interictal ghrelin levels in adults with focal and generalized epilepsy. AG: acyl ghrelin; ASD: antiseizure drug; CBZ: carbamazepine; DAG: desacyl ghrelin; DR-TLE: drug-resistant temporal lobe epilepsy; PHT: phenytoin; Ref: reference; TLE: temporal lobe epilepsy; VPA: valproic acid. * the different ghrelin levels in epilepsy patients versus controls. ** the differences in generalized epilepsy versus focal epilepsy.

ASD	Ghrelin Form	Controls (pg/mL)	*	Epilepsy Patients (pg/mL)		**	Ref
				Focal Seizures	Generalized Seizures		
VPA, PHT, CBZ	Total	93	↑	234	134	↓	[85]
VPA, PHT, CBZ	DAG AG	DAG: 585 AG: 46	↓	DAG: 439 AG: 35	DAG: 267 AG: 23	↓	[80]
VPA, PHT, CBZ	Total	700	↓	500	300	↓	[81]
VPA	N/A	381	=	364 (both types)		/	[83]
VPA, CBZ	DAG AG	DAG: 196 AG: 7	=	DAG: 207-239, AG: 7-22	DAG: 250 AG: 8	=	[84]
N/A	DAG AG	DAG 242 AG: 13	↑	DAG: 238 AG: 14.5	DAG: 245 AG: 19	↑	[86]
N/A	N/A	1320	↓	TLE: 1010	DR-TLE: 910	/	[82]

**Table 2:** Overview of interictal ghrelin levels in children. AG: acylated ghrelin; ASD: antiseizure drug; CBZ: carbamazepine; TPM: topiramate; DAG: desacyl ghrelin; Ref: reference; VPA: valproic acid. * the different ghrelin levels in epilepsy patients versus controls. ** the difference in condition 2 versus condition 1 within epilepsy patients. Age denotes either the mean age of the patient groups rounded to the nearest integer, or the age range.

ASD	Ghrelin Form	Controls (pg/mL)	*	Epilepsy Patients (pg/mL)		**	Age (Years)	Ref
VPA, CBZ	Total	554	↓	VPA: 381	CBZ: 283	↓	5	[91]
VPA, TPM	N/A	267	=	VPA: 240	TPM: 267	=	6–15	[94]
VPA	DAG AG	DAG: 446 AG: 45	↓	Pretreatment: DAG: 420 AG: 36	Post-treatment: DAG: 459 AG: 51	↑	9	[93]
VPA	N/A	333	↓	Pretreatment: 355	Post-treatment: 263	↓	11	[92]

**Table 3:** Overview of interictal ghrelin levels in children after ASD or KD intervention. AG; acylated ghrelin; ASD: antiseizure drug; CBZ: carbamazepine; d: day; DAG: desacyl ghrelin; Int: intervention; KD: ketogenic diet; LEV: levetiracetam; m: month; OXC: oxcarbazepine; PHT: phenytoin; Ref: reference; T: time; TPM: topiramate; VPA: valproic acid; y: year. * the different ghrelin levels in epilepsy patients over time. ± the concentrations derived from graphs.

Int.	Ghrelin Form	Baseline (pg/mL)	T1	T2	T3	T4	*	Age (Years)	Ref
OXC	N/A	327	6 m: 306	18 m: 320	/	/	=	9	[95]
OXC	N/A	310	6 m: 288	18 m: 345	/	/	=	13	[98]
VPA	N/A	18	6 m: 18	12 m: 18	/	/	=	9	[96]
VPA	N/A	334	6 m: 275	18 m: 245	/	/	↓	14	[98]
VPA	N/A	1.37	6 m: 2.19	/	/	/	↑	8	[99]
TPM	N/A	1121	3 m: 1184	6 m: 1292	/	/	=	8	[97]
LEV	N/A	1900	6 m: 2950	/	/	/	=	7	[100]
KD	DAG AG	DAG: ±160 AG: ±250	15 d: DAG: ±110 AG: ±210	30 d: DAG: ±100 AG: ±140	90 d: DAG: ±140 AG: ±110	/	↓	7	[101]
KD	AG	±400	15 d: ±250	30 d: ±200	90 d: ±200	1 y: ±200	↓	6	[102]
KD	N/A	20	6 m: 19	12 m: 19	/	/	/	8	[103]

**Table 4:** Overview of effects of ghrelin in experimental epilepsy models. i.c.v.: intracerebroventricular; i.h.: intrahippocampal; IHKA: intrahippocampal kainic acid; i.p.: intraperitoneal; KA: kainic acid; min: minute; pen: penicillin; pilo: pilocarpine; PTZ: pentylenetetrazole; Ref: reference.

Dose	Administration Regimen	Anticonvulsant	Animal Model	Ref
0.02–0.08 mg/kg	i.p. 30 min prior to PTZ	yes	PTZ i.p. rat model	[105,117]
0.08 mg/kg	i.p. 30 min prior to PTZ	no	PTZ i.p. rat model	[118]
0.3 nmol/µL	i.h. infusion 1 x 30 min prior to PTZ or 10 days	yes	PTZ i.p. rat model	[106,107]
0.08 mg/kg	i.c.v. 30 min prior to PTZ	yes	PTZ i.p. rat model (female rats)	[108]
0.5, 1 and 2 µg	i.c.v. 30 min after pen	yes	Intracortical penicillin rat model	[113,119]
0.08 mg/kg	i.p., immediate assessment	no	WAG/Rij rat model	[114]
0.01–10 µM	i.h. infusion, 120 min prior to pilo	yes	Pilocarpine i.h. infusion rat model	[116]
1.5 mg/kg	i.p. 10 min prior to pilo	no	Pilocarpine i.p. rat model	[31,115]
1.5 mg/kg	i.p. 10 min prior to KA	no	KA i.p. rat model	[115]
0.08 mg/kg	i.p. 30 min prior to KA, and 24 h after KA	yes	KA i.p. mouse model	[29]
1.8 mg/kg	i.p. 30 min prior to pilo	yes	Pilocarpine tail infusion mouse model	[116]

**Table 5:** Overview of effects of desacyl ghrelin in experimental epilepsy models. i.c.v.: intracerebroventricular; i.p.: intraperitoneal; KA: kainic acid; min: minute; pilo: pilocarpine; Ref: reference.

Desacyl Ghrelin				
Dose	Administration Regimen	Anticonvulsant	Animal Model	Ref
1.5 mg/kg	i.p. 10 min prior to pilo	yes/no (*p* = 0.07)	Pilocarpine i.p. rat model	[115]
1.5 mg/kg	i.p. 10 min prior to KA	yes	KA i.p. rat model	[115]
1–10 µM	i.c.v. 2 h prior to pilo	yes	Pilocarpine i.c.v. rat model	[37]
3/5 mg/kg	i.p. 30 min prior to pilo	yes	Pilocarpine tail infusion mouse model	[37]
